# Room Temperature Characteristics of Polymer-Based Low Ice Adhesion Surfaces

**DOI:** 10.1038/srep42181

**Published:** 2017-02-07

**Authors:** Zhiwei He, Elisabeth T. Vågenes, Chrisrosemarie Delabahan, Jianying He, Zhiliang Zhang

**Affiliations:** 1 NTNU Nanomechanical Lab, Department of Structural Engineering, Norwegian University of Science and Technology (NTNU), Trondheim 7491, Norway

## Abstract

Ice adhesion is mainly dictated by surface properties, and water wettability is frequently correlated with ice adhesion strength. However, these established correlations are limited to high ice adhesion and become invalid when the ice adhesion strength is low. Here we carried out an experimental study to explore the relationships between low ice adhesion strength and room temperature surface properties. A variety of room temperature properties of 22 polymer-based hydrophilic and hydrophobic samples consisting of both low and high ice adhesion surfaces were analysed. The properties investigated include water adhesion force, water wettability, roughness, elastic modulus and hardness. Our results show that low ice adhesion strength does not correlate well with water contact angle and its variants, surface roughness and hardness. Low elastic modulus does not guarantee low ice adhesion, however, surfaces with low ice adhesion always show low elastic modulus. Low ice adhesion (below 60 kPa) of tested surfaces may be determinative of small water adhesion force (from 180 to 270 μN). Therefore, measurement of water adhesion force may provide an effective strategy for screening anti-icing or icephobic surfaces, and surfaces within specific values of water adhesion force will possibly lead to a low ice adhesion.

The inevitable accretion of ice on exposed surfaces severely influences many normal industrial operations and activities, such as energy distribution, transportation, aircraft, and offshore platforms^1^,^2^,^3^. To make operational performance safe and effective, accreted ice is usually mitigated by using deicing liquids or salts and thermal energy^4^,^5^. These suboptimal approaches not only induce high costs but also have detrimental effects on the environment. An appealing strategy is to introduce passive icephobic coatings to repel ice or reduce ice adhesion on the exposed surfaces. Although it is difficult to keep surfaces free of ice, hydrophilic, hydrophobic and superhydrophobic surfaces have been demonstrated to be effective in reducing ice adhesion over the past decades^6^,^7^,^8^. Recently, there is a surging interest in the elucidation of relationships between ice adhesion and room temperature characteristics, which will provide design principles for the development of icephobic surfaces. In the literature, water contact angle as well as its variants is one characteristic that has been claimed to correlate with ice adhesion. In particular, the value of [1 + cos *θ*_rec_] is shown to correlate well with ice adhesion strength (larger than 160 kPa)^2^. However, some questions still remain elusive regarding this strong correlation: (1) the validity for surfaces with low ice adhesion and (2) the validity for elastomeric surfaces. Despite the fact that low ice adhesion is one of the main goals for icephobic surfaces utilized in harsh environments^9^, evidence of such a correlation for surfaces with low ice adhesion is still missing. Wang *et al*. reported that the ice adhesion of the elastomeric PDMS nanocomposite was largely dependent on coating thickness^10^, while we in our recent experiments found that the value of [1 + cos *θ*_rec_] was almost the same for the PDMS surfaces with different layer thickness^11^. Durable PDMS based icephobic surfaces with similar values of [1 + cos *θ*_rec_], as designed by Golovin *et al*., also showed large variation for ice adhesion strength^7^. To further understand and explain these differences, better correlations between ice adhesion strength and different room temperature characteristics are needed.

Water wettability is a commonly accepted parameter that correlates with ice adhesion strength. A surface with a higher water contact angle (WCA) is considered to have a lower ice adhesion strength because of the low surface energy for both water and ice^12^. Sarkar *et al*. and other groups demonstrated that the surfaces with higher WCA, especially for superhydrophobic surfaces, showed lower ice adhesion^1^,^13^,^14^,^15^,^16^,^17^. In addition to high WCA, the large initial size of interfacial cracks^18^,^19^ and low wetting hysteresis associated with the ice-solid contact area^20^ also contribute to low ice adhesion strength. The reason why high WCA is not adequate to yield low ice adhesion strength is possibly due to the different dynamic processes^2^. Sliding angle (SA) and contact angle hysteresis (CAH) have been shown to correlate with ice adhesion strength, indicating a better correlation with ice adhesion strength than the conventional WCA^20^,^21^,^22^,^23^,^24^,^25^,^26^,^27^. This is because there is a shearing process caused by the movement of a droplet on a surface when SA and CAH are measured^28^,^29^. Besides, further studies showed that CAH does not always correlate well with ice adhesion strength^30^,^31^. Recently, Meuler *et al*. treated the wettability from a thermodynamic viewpoint and considered the free energies associated with the formation and elimination of interfacial areas^2^,^32^. For example, the reversible free energy associated with creation and destruction of interfaces (*W*_e_)^33^ and the actual work required to separate a liquid from a surface (*W*_p_)^2^,^34^ are linked to the equilibrium contact angle and receding contact angle by the following two equations^2^,^28^,^29^:


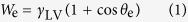



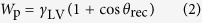


where *θ*_e_ is the equilibrium contact angle, *θ*_rec_ is the receding contact angle, and *γ*_LV_ is the liquid-vapor interfacial tension. Meuler *et al*. showed a strong linear relationship between the value of [1 + cos *θ*_rec_] and ice adhesion strength (larger than 160 kPa) for both hydrophilic and hydrophobic surfaces^2^. This linear correlation has been confirmed by others^35^,^36^,^37^. The correlation for low ice adhesion surfaces, however, has not been explored.

Roughness is also associated with ice adhesion strength. It was reported that reduced roughness tends to reduce ice adhesion^6^,^30^,^35^,^38^,^39^,^40^,^41^. Smooth surfaces, such as surfaces with aqueous lubricating layers^6^,^38^ and slippery liquid-infused porous surfaces (SLIPS)^39^, showed low ice adhesion strength. Furthermore, Davis *et al*.^30^ and Zhu *et al*.^40^ also showed that reduced roughness resulted in the reduction of ice adhesion strength, which corresponds to other results showing that reduced roughness decreases the inter-locking effect between ice and substrate^25^,^42^,^43^. On the contrary, however, Liu *et al*. and Farhadi *et al*. demonstrated that a decrease in roughness led to an increase in ice adhesion strength^44^,^45^. Therefore, roughness may not be uniquely linked to ice adhesion strength.

Water adhesion force is a surface characteristic not yet explored which might demonstrate a correlating factor to ice adhesion strength. Water adhesion force is measured from the detaching process of a water droplet from the substrate which is similar to the ice detaching process. Water adhesion reflects both the surface chemical composition and the contact area^46^. As for the surface chemistry, there are three interactions on a molecular scale, including the electrostatic attraction, covalent bonding and van der Waals forces. Particularly, the electrostatic attraction between water/ice and a solid surface is significant, and the attraction of water is similar to that of ice since water and ice are both polar materials^17^. As for the contact area, possible inter-locking effects between ice and micro/nanostructures can increase ice adhesion if water penetrates into the micro/nanostructured surfaces before it freezes. Recently, it has been proven by many groups that changes in surface chemistry and contact area can be accurately detected by changes in water adhesion force^46^,^47^. Furthermore, the potential surface deformation can be measured for surfaces with low elastic modulus during the detachment of a water droplet^48^. Other parameters, such as water wettability and roughness, however, cannot characterize the potential surface deformation for surfaces with low elastic modulus. Water adhesion force may be a potential parameter that governs low ice adhesion.

Although low ice adhesion surfaces play a significant role in various industries, high development cost and the difficulty of low temperature evaluation of these surfaces hinder their development. Effective correlations between low ice adhesion strength and room temperature surface characteristics would be extremely valuable. Herein, we examine relationships between low ice adhesion strength and various room temperature properties for 22 polymer-based hydrophilic and hydrophobic coatings, such as water adhesion force, roughness, elastic modulus, hardness, and water contact angle as well as its variants. Our results show that low ice adhesion of tested surfaces may be determinative of small water adhesion force (from 180 to 270 μN), which may provide an effective strategy for screening anti-icing or icephobic surfaces by evaluation of water adhesion force at room temperature.

## Results and Discussion

### Correlations between low ice adhesion strength and wettability

Table 1 shows the measurements of water contact angle, roughness, water adhesion force, and ice adhesion strength on 22 polymer-based coatings (S1–S22), bare aluminum (S23) and bare steel (S24). All the samples (S1–S22) (Fig. S1) are prepared by four commercial companies, and they are used as received to characterize the room temperature characteristics and ice adhesion as described in two Master theses^49^,^50^. The exact chemical compositions are proprietary and were not disclosed. Table S1 in supplementary information describes the types of surface components of most coatings. In our study, ice adhesion strength less than 60 kPa is defined as low ice adhesion strength. It can be found in Table 1 that ice adhesion strength ranges from 6.88 kPa to 714 kPa, and there are 17 samples with low ice adhesion. Bare aluminum and steel surfaces were tested solely to give reference values of ice adhesion. The ice adhesion strengths for bare Al and steel surfaces are 487 kPa and 714 kPa, which are comparable to the values in other literature^2^,^51^. Schematic illustrations of the working principles for the ice detachment, the design of the ice adhesion test apparatus, and the method of ice formation are explained in the Supplementary Information Figs S2–5, respectively. The focus of this study is to investigate the relationship between low ice adhesion strength and room temperature surface characteristics on polymer-based surfaces.

**Figure 1:**
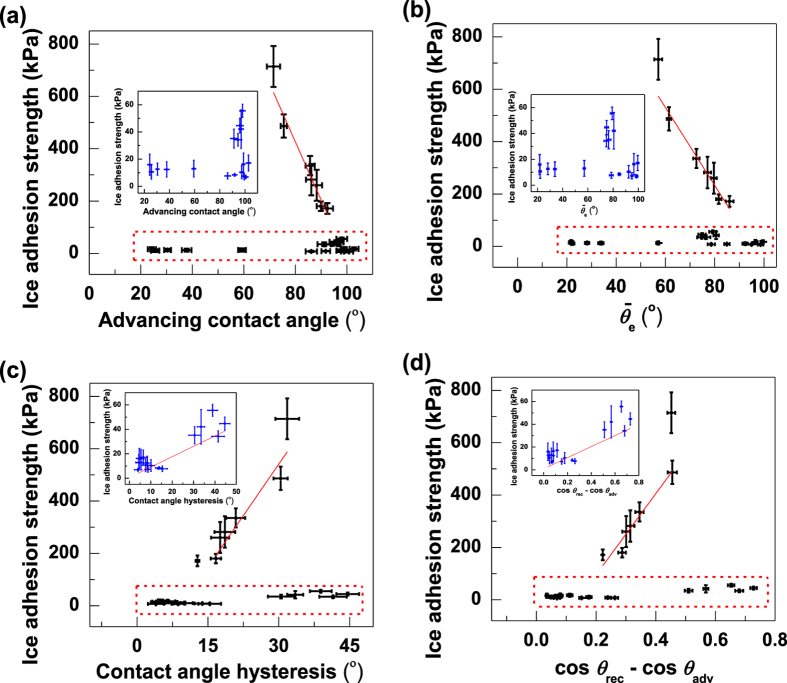
Average ice adhesion strength of the 24 samples plotted against two values of WCA and two values of CAH. (**a**) the water advancing contact angle *θ*_adv_, (**b**) the estimated equilibrium contact angle 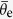, (**c**) *θ*_rec_ − *θ*_adv_, and (**d**) cos *θ*_rec_ − cos *θ*_adv_. The inserted sub-figures present the data in the highlighted areas. Uncertainties of wettability and ice adhesion are standard deviations for the 5 measurements.

In order to examine the relationship between ice adhesion strength and water wettability, the ice adhesion strength is plotted against two values of WCA and two values of CAH as shown in Fig. 1. It is difficult to measure the equilibrium static WCA (*θ*_e_), and the 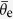
can be estimated from the measurements of *θ*_adv_ and *θ*_rec_, by using the following equation^2^,^52^,^53^:


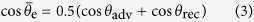


Figure 1a and b show that ice adhesion strength has a linear correlation with *θ*_adv_ (the square of the correlation coefficient, R^2^ = 0.92) and 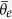
(R^2^ = 0.90) when the ice adhesion strength is larger than 60 kPa. This result indicates that ice adhesion strength decreases as the WCA increases, which is in agreement with previous studies^17^,^24^,^41^,^54^,^55^,^56^. However, the advancing contact angles and estimated equilibrium static WCA of surfaces with low ice adhesion spread from 20° to 110° as shown in the highlighted areas and the inserted sub-figures of Fig. 1a and b, indicating its independence on WCA. In Fig. 1c and d, CAH in different forms of [*θ*_adv_ − *θ*_rec_] and [cos *θ*_rec_ − cos *θ*_adv_] is used to correlate with ice adhesion strength. For the coatings with high ice adhesion strength, R^2^ for [*θ*_adv_ − *θ*_rec_] and [cos *θ*_re_ − cos *θ*_adv_] are 0.90 and 0.74. These results show that [*θ*_adv_ − *θ*_rec_] correlates better with ice adhesion strength than [cos *θ*_rec_ − cos *θ*_adv_], similar to previous results^30^,^31^. For the coatings with low ice adhesion strength, R^2^ for [*θ*_adv_ − *θ*_rec_] and [cos *θ*_rec_ − cos *θ*_adv_] are 0.64 and 0.59 as shown in the inserted sub-figures of Fig. 1c and d. These results reveal that [*θ*_adv_ − *θ*_rec_] and [cos *θ*_rec_ − cos *θ*_adv_] show a weak linear relationship with low ice adhesion strength. The above results indicate that the linear trends for high and low ice adhesion strength are different.

**Figure 2:**
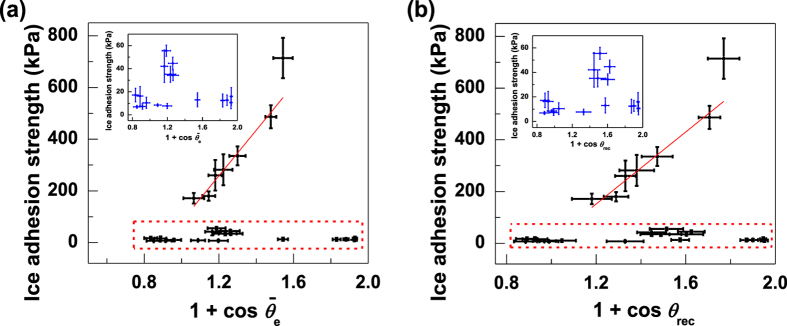
Average ice adhesion strength of the 24 samples plotted against [1 + cos 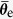] and [1 + cos *θ*_rec_]. (**a**) the equilibrium work of adhesion for liquid water (see equation 1), and (**b**) the practical work of adhesion for liquid water (see equation 2). The inserted sub-figures present the data in the highlighted areas. Uncertainties of wettability and ice adhesion are standard deviations for the 5 measurements.

In Fig. 2, ice adhesion strength is plotted with the values of (1 + cos 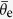) and (1 + cos *θ*_rec_) for all samples tested including the bare Al and steel. The correlations with [1 + cos 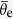] (R^2^ = 0.88) and [1 + cos *θ*_rec_] (R^2^ = 0.86) are similar for samples with high ice adhesion strength. Furthermore, low ice adhesion strengths of the 17 polymer-based coatings do not correlate well with [1 + cos *θ*_rec_] (or [1 + cos 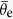]) as illustrated in the highlighted areas and the inserted sub-figures of Fig. 2a and b. With the similar value of [1 + cos *θ*_rec_] (e.g., 1.2), ice adhesion strength even varies from 7.7 kPa to 282 kPa for different samples. Low ice adhesion strength is shown to be independent of the values of [1 + cos *θ*_rec_] (or [1 + cos 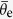]) in these tested samples (S1–S17). The validity for a correlation between low ice adhesion and the value of [1 + cos *θ*_rec_] needs to be further investigated.

### Correlations between low ice adhesion strength and water adhesion force, roughness, elastic modulus and hardness

**Figure 3:**
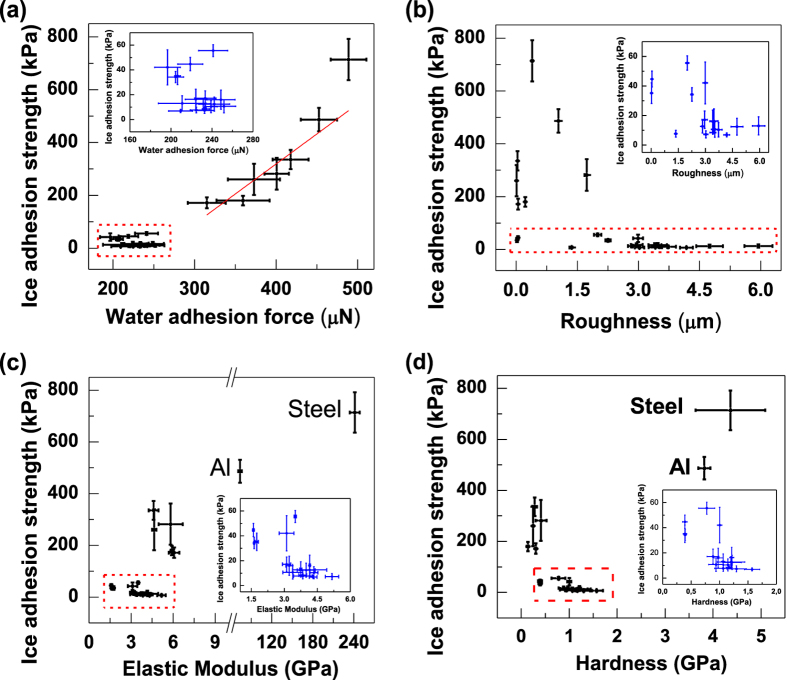
Average ice adhesion strength plotted against water adhesion force, roughness, elastic modulus and hardness. (**a**) water adhesion force, (**b**) roughness, (**c**) elastic modulus and (**d**) hardness. The inserted sub-figures present the data in the highlighted areas. Uncertainties of water adhesion force, roughness and elastic modulus (or hardness) are standard deviations for the 10, 5 and 9 measurements, respectively.

In Fig. 3a, high ice adhesion strengths of the 7 samples reveal a linear correlation with water adhesion forces, while the 17 samples with low ice adhesion strengths possess much lower water adhesion forces as shown in the highlighted area and the inserted sub-figure of Fig. 3a. The results show that low ice adhesion strength is associated with low water adhesion force ranging from 180 to 270 μN. Similar to the ice adhesion, the value of water adhesion force represents the capability to detach the water droplet beyond surface barriers and hindrances. As revealed in Fig. 3b–d and Supplementary Table S2, both high and low ice adhesion strengths do not correlate well with roughness, elastic modulus and hardness for the 24 samples (S1–S24). It can be observed that low elastic modulus does not guarantee low ice adhesion, while surfaces with low ice adhesion strength always show low elastic modulus (Fig. 3c). Furthermore, it is interesting to note that the elastic modulus of all the low ice adhesion surfaces is smaller than that of ice (about 9 GPa)^57^, which indicates that low ice adhesion occurs when the surface is under-matched by the ice.

The wettability variant, like the value of [1 + cos *θ*_rec_], is unable to characterize potential surface deformation for surfaces with low elastic modulus during measurements. This may be a possible reason to explain why surfaces with similar value of [1 + cos *θ*_rec_] have quite different ice adhesion strengths. For the 17 hydrophilic or less hydrophobic surfaces with low elastic modulus, noticeable surface deformation occurs during the measurements of water adhesion forces as shown in Supplementary Figure S6, which is in analogous to the surface deformation induced for surfaces with low elastic modulus during the detachment of ice. This means the detachments of water droplets on surfaces need to simultaneously overcome not only the resistance of surface topographies and chemistry, but also potential surface deformations. Therefore, water adhesion forces may be more accurate than wettability variants to correlate with ice adhesion strength for surfaces with low elastic modulus.

To conclude, ice adhesion strengths of 24 surfaces were measured to compare the correlations with other surface characteristics such as water wettability, roughness, water adhesion forces, elastic modulus and hardness. Our results show that low ice adhesion strength may be determinative of small water adhesion force (from 180 to 270 μN), and does not correlate well with other room temperature characteristics. Similar to the ice detachment tests, water adhesion forces may be able to characterize the potential surface deformation process. Water adhesion force could be therefore used to estimate low ice adhesion at room temperature, which greatly facilitates the design and fabrication of low-cost anti-icing or icephobic surfaces.

## Methods

### Materials

Five hydrophobic (S1, S2, S5, S11, S12) and seventeen hydrophilic (S3, S4, S6–S10, S13–S22) polymer based coatings, aluminum (S23), and steel (S24) were received from four commercial companies as described in two Master Theses^49^,^50^. The exact chemical compositions of these coatings are proprietary and were not disclosed. Table S1 in supplementary information describes the types of surface components of most coatings. Deionized (DI) water with a resistivity of 18.2 MΩ·cm^−1^ was used for the measurements of contact angles and water adhesion forces.

### Characterization

The water contact angles of different samples were measured by using a CAM 200 contact-angle system at room temperature. Advancing (*θ*_adv_) and receding (*θ*_rec_) angles were measured as water was supplied through a syringe into or out of sessile droplets (~5 μL) and repeated 5 times. The water adhesion forces were investigated by Dynamic Contact Angle Tensiometer (dataphysics DCAT11®) at room temperature and each substrate was tested and repeated 10 times^58^,^59^. The roughness of representative test samples was measured using a contact stylus profilometer (Dektak 150) equipped with a 12.5 μm diamond tip stylus and repeated 5 times. The elastic modulus and hardness were obtained from the measurements of nanoindentation (TriboIndenter^®^ 950, Hysitron, Inc.) and repeated 9 times.

### Measurement of ice adhesion strength

The ice adhesion test was modified from a laboratory ice adhesion test^60^. The water was frozen at −18 °C in the polypropylene (PP) tube molds for 24 hours as ice cylinders. A force probe with the diameter of 3 mm propelled the tube-encased ice columns at a velocity of 0.1 mm·s^−1^, and the probe was located close to the test sample surface (less than 1 mm) to minimize torque on the ice sample. Moreover, a schematic illustration of the working principle for ice detachments, the details of ice formation molds and the design of the sample holder are provided in the Supplementary Information. Uncertainties of ice adhesion are standard deviations for the 5 measurements.

## Additional Information

**How to cite this article:** He, Z. *et al*. Room Temperature Characteristics of Polymer-Based Low Ice Adhesion Surfaces. *Sci. Rep.* **7**, 42181; doi: 10.1038/srep42181 (2017).

**Publisher's note:** Springer Nature remains neutral with regard to jurisdictional claims in published maps and institutional affiliations.

## Supplementary Material

Supplementary Information

## Figures and Tables

**Table 1:** Measured water contact angle, roughness, water adhesion force and average strength of ice adhesion for the 24 tested surfaces.

Samples	*θ*_adv_	*θ*_rec_	Root-mean square roughness *R*_q_ (μm)	Water adhesion force (μN)	Ice adhesion strength (kPa)
S1	100.6 ± 1.8	96.6 ± 1.5	4.177 ± 0.159	209.4 ± 11.6	6.880 ± 1.26
S2	99.3 ± 1.6	90.6 ± 1.2	3.025 ± 0.115	232.8 ± 9.14	7.258 ± 2.36
S3	86.2 ± 2.2	70.8 ± 2.5	1.362 ± 0.0572	224.7 ± 5.96	7.677 ± 2.28
S4	91.8 ± 1.7	78.0 ± 2.5	3.414 ± 0.164	234.1 ± 3.52	8.443 ± 1.05
S5	97.4 ± 1.5	87.3 ± 1.8	3.726 ± 0.194	239.3 ± 6.17	10.38 ± 4.85
S6	25.6 ± 1.6	18.0 ± 1.5	3.520 ± 0.187	251.7 ± 11.3	10.65 ± 5.34
S7	37.8 ± 1.9	29.4 ± 1.6	4.746 ± 0.327	242.2 ± 14.9	12.40 ± 5.66
S8	30.4 ± 1.3	25.9 ± 1.5	2.839 ± 0.0965	231.5 ± 6.87	12.66 ± 4.46
S9	59.3 ± 1.5	54.9 ± 1.4	5.944 ± 0.345	210.9 ± 23.2	13.00 ± 6.11
S10	24.3 ± 1.6	18.9 ± 1.2	3.404 ± 0.160	248.7 ± 13.7	15.95 ± 7.73
S11	98.8 ± 1.2	94.2 ± 1.6	3.471 ± 0.226	224.2 ± 10.2	16.26 ± 8.18
S12	102.8 ± 1.7	96.3 ± 1.4	2.957 ± 0.145	233.4 ± 10.4	17.14 ± 5.87
S13	94.4 ± 3.1	52.9 ± 2.7	2.255 ± 0.0654	204.5 ± 7.80	34.24 ± 4.56
S14	91.2 ± 2.6	60.7 ± 3.0	0.01957 ± 0.000881	206.4 ± 2.75	35.14 ± 6.99
S15	96.9 ± 1.8	63.4 ± 1.6	2.990 ± 0.123	196.6 ± 12.5	42.07 ± 14.1
S16	95.9 ± 2.6	51.3 ± 2.2	0.04294 ± 0.00155	218.8 ± 12.1	44.67 ± 5.34
S17	98.0 ± 2.1	59.1 ± 2.5	1.999 ± 0.090	241.1 ± 14.1	55.53 ± 4.83
S18	92.4 ± 2.2	79.6 ± 2.6	0.04608 ± 0.00272	315.0 ± 23.3	171.7 ± 20.3
S19	89.9 ± 1.6	73.2 ± 1.9	0.2225 ± 0.0102	359.4 ± 32.6	180.1 ± 18.2
S20	88.3 ± 2.1	70.7 ± 1.7	0.0105 ± 0.00662	372.8 ± 31.9	260.5 ± 79.0
S21	86.2 ± 2.1	67.6 ± 2.3	1.732 ± 0.0554	400.7 ± 15.0	282.0 ± 79.9
S22	82.7 ± 1.8	61.8 ± 2.2	0.03479 ± 0.00157	417.8 ± 22.0	335.5 ± 36.4
S23	75.5 ± 1.2	45.1 ± 1.7	1.036 ± 0.0591	452.6 ± 22.3	486.8 ± 44.3
S24	71.5 ± 2.6	39.7 ± 2.4	0.3934 ± 0.0181	488.8 ± 21.9	714.0 ± 78.0
