# Supplementary material for: Room Temperature Characteristics of Polymer-Based Low Ice Adhesion Surfaces
Source: Sci Rep. 2017 Feb 7;7:42181. doi: 10.1038/srep42181 (PMC5294566; doi:10.1038/srep42181)
Supplement: Supplementary Information [file srep42181-s1.pdf]

Room Temperature Characteristics of Polymer-based Low Ice Adhesion Surfaces

Zhiwei He,[a] Elisabeth T. Vågenes,[a,b,#] Chrisrosemarie Delabahan,[a] Jianying He[a] & Zhiliang Zhang[a]*

[a]NTNU Nanomechanical Lab, Department of Structural Engineering, Norwegian University of Science and Technology (NTNU), Trondheim 7491, Norway

[b]SINTEF Energy Research, Gas Technology, Sem Sælands vei 11, 7034 Trondheim, Norway.

[#]Elisabeth T. Vågenes is now working at SINTEF.

Correspondence and requests for materials should be addressed to Z.Z. (email: zhiliang.zhang@ntnu.no)

Supplementary Figures S1-S6 and Table S1-S2

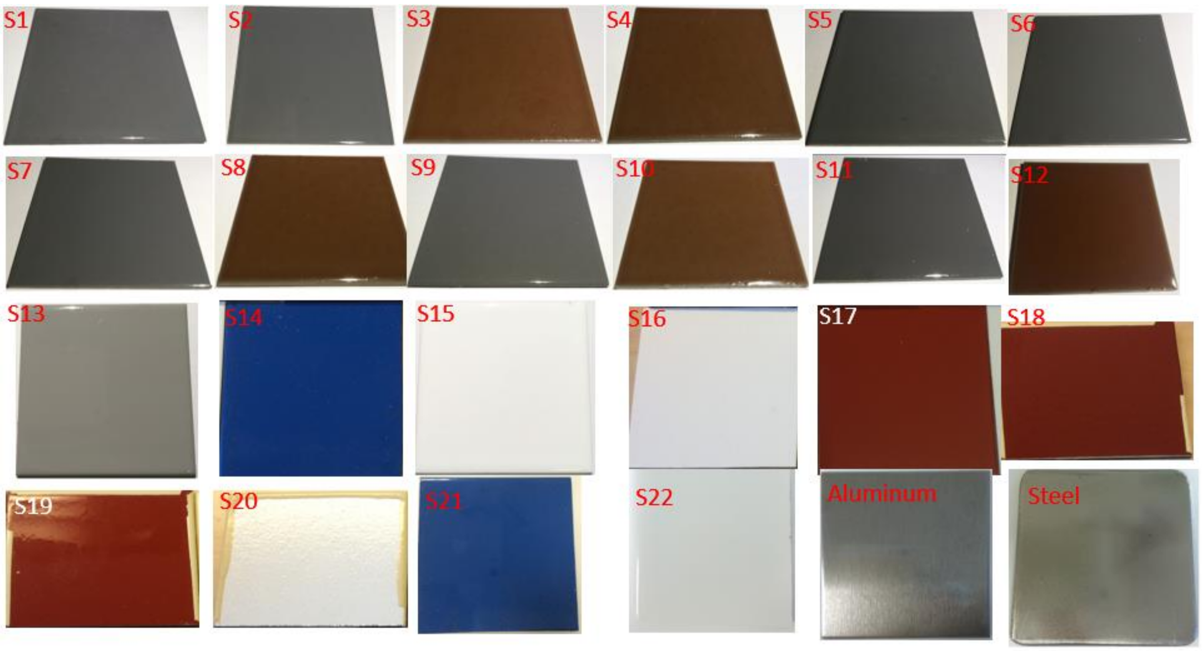

Figure S1. Photographs of polymer-based coatings (S1-S22) and the reference samples (aluminum and steel).

Table S1 describes the components of PDMS-based coatings (S1-S12). The samples (S13, S14 and S16) are silicone resin based coatings. The samples (S15 and S17) are multilayer coatings based on commercial paints. The components of samples (S18-S22) cannot be revealed.

Samples		S1	S2	S3	S4	S5	S6	S7	S8	S9	S10	S11	S12
Base	PDMS	×	×	×	×	×	×	×	×	×	×	×	×
Filler	Nanoscale amorphous silica	×								×		×	
	Superhydrophobic nanoparticles		×	×	×	×	×	×	×		×		
Additive	Non-reactive hydrophobic additive	×	×	×	×	×							
	Non-reactive hydrophilic additive						×	×	×	×	×		
Treatment	Treated with fluoride				×	×	×		×				

Table S1. The components of PDMS-based coatings (S1-S12). The components of samples (S1-S12) are discussed, including base, filler, additive and fluoride. The symbol “×” means “has the component or treatment”. Additives are used for the purpose of improving hydrophilicity or hydrophobicity.

A schematic illustration of the working principle for ice detachment is described in Figure S2.

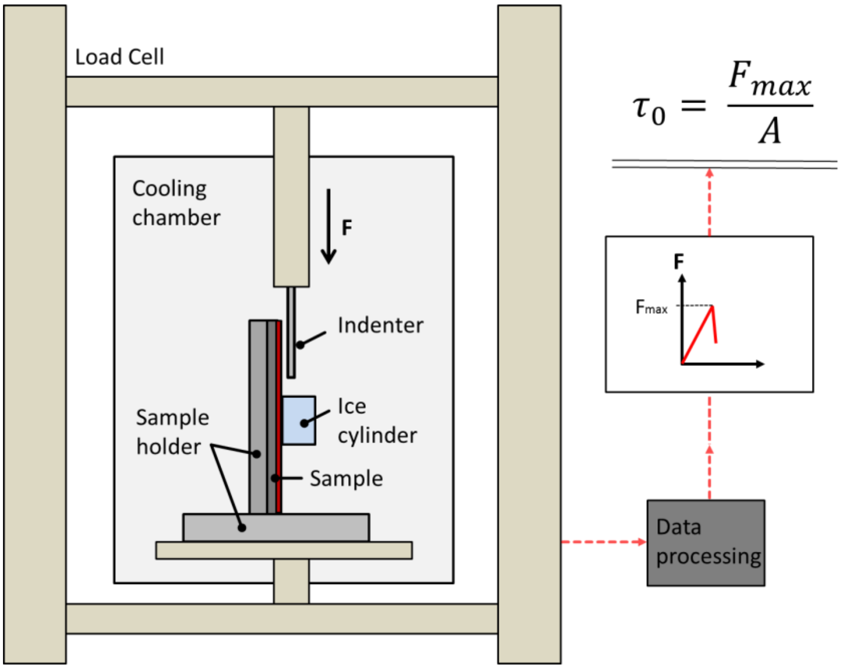

Figure S2. Schematic illustration of the working principle for the proposed method. A load cell with an indenter applies a load to an ice cylinder frozen onto a fixed test samples. The normal load induces a shear stress in the ice/solid interface, which can be calculated from the recorded data. The ice adhesion strength (τ_0) equals the shear strength, defined as the maximum force (F_{max}) divided by the ice/solid contact area (A).

1 Design of the apparatus

1.1 The sample holder

The sample holder is designed to keep the samples fixed vertically inside the chamber during testing. It can support a sample with a width between 6 and 10 cm and a thickness up to 0.8 cm (Figure S3). The sample holder consists of a stainless steel plate for the supporting block, brass plates for the upper parts, and socket head cap screws.

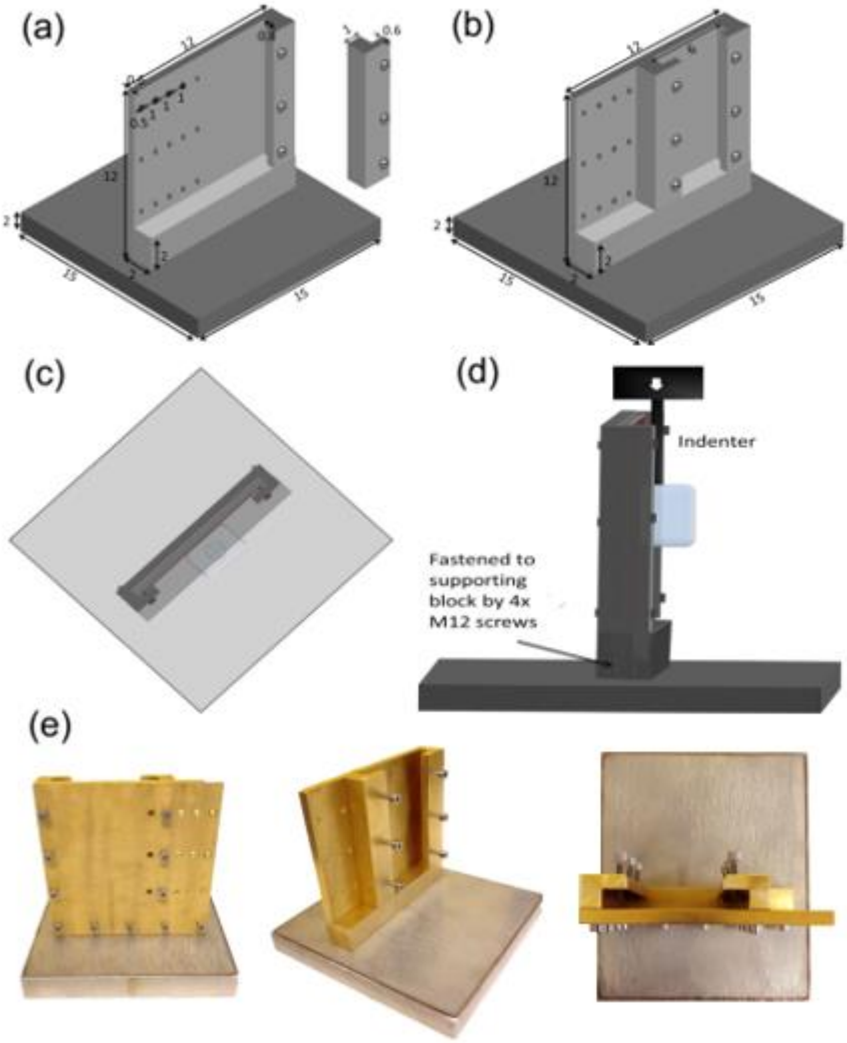

Figure S3. Schematic images of the sample holder. (a) front view; (b) adjusted for a 6×6 cm sample; (c) top view; (d) side view; (e) manufactured sample holder seen from different views.

1.2 The load cell and chamber

In Figure S4a, a cooling chamber (Zwick, WT 160 LN2) is mounted around the Zwick/Roell Z030, which has an inlet for liquid nitrogen and a digital temperature display (Eurotherm 2408).

2 The formation of the ice cylinders

In general, there are two criterions for molds of this ice formation method: (a) a small contact surface without sharp edges; (b) universally available. The 50 mL polypropylene (PP) centrifuge tubes with a wall of 1 mm thick and an inner diameter of 27.5 mm are chosen as molds for the ice formation (Figure S5a). Firstly, the PP tubes are cut off from the top, and the upper rim of this tube is used as the contact edge between the ice and the testing sample because of its regularity. Then, a thin layer of Loctite 5926 Silicone Blue sealant that remains soft in a temperature range from -55 ºC to 200 ºC (Figure S5c) is applied to PP tubes to avoid water leakage, followed by some pressure of a 200 g metal cylinder (Figure S5b and Figure S5d). Finally, 10 mL distilled water is carefully injected into the tubes by a plastic syringe without any visible air bubbles and frozen for 24 hours as shown in Figure S5d.

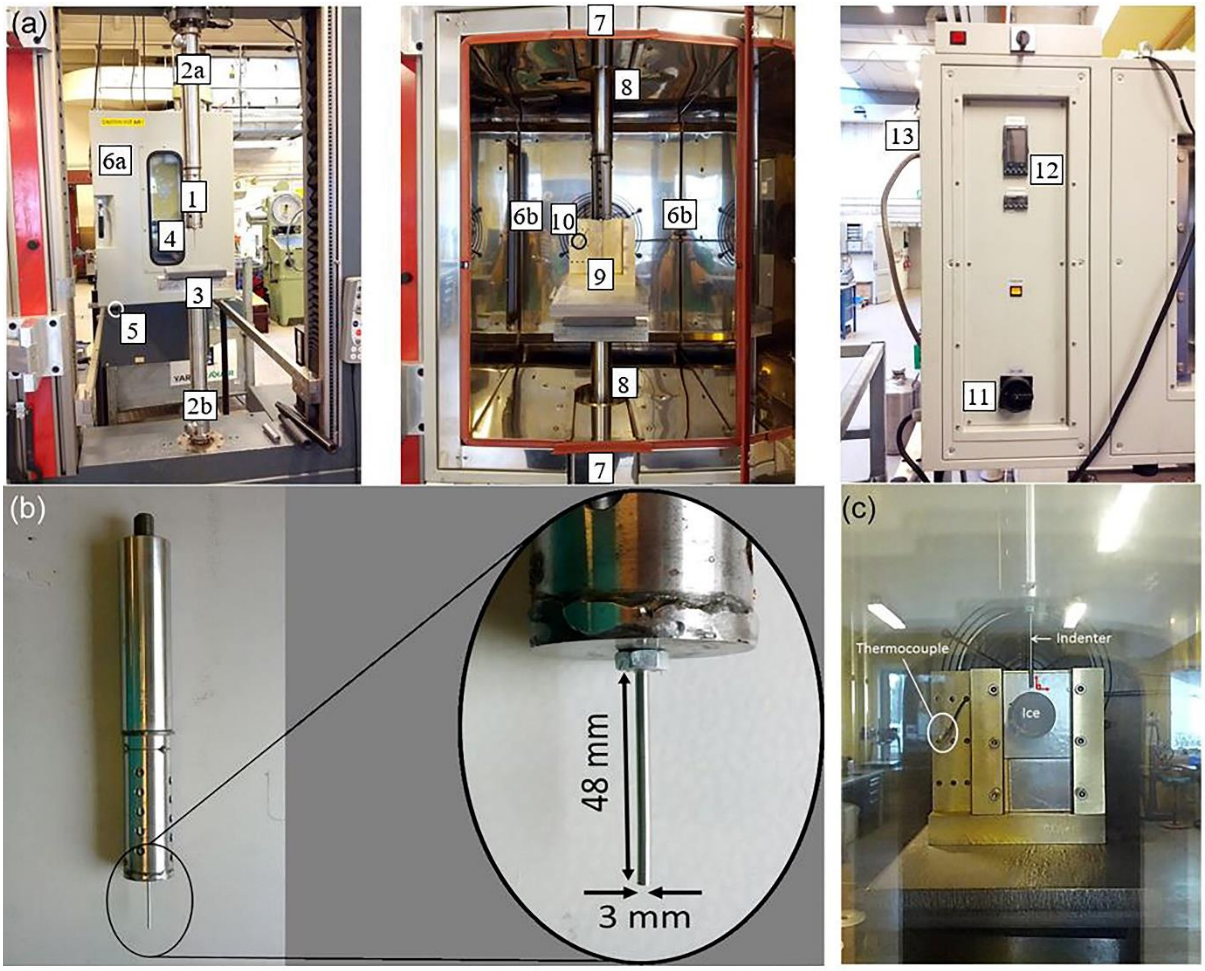

Figure S4. The instrument for ice adhesion measurements. (a) Images with numbered items of this testing instrument: (1) indenter; (2) upper and lower mounting stud; (3) Zwick fixture; (4) observation window; (5) switch; (6) cooling chamber; (7) removable parts; (8) openings in the top and bottom; (9) sample holder; (10) thermocouple wire; (11) on-off button; (12) temperature setting; (13) inlet hose. (b) The indenter fastened onto the existing Zwick fixture. (c) The chamber during testing.

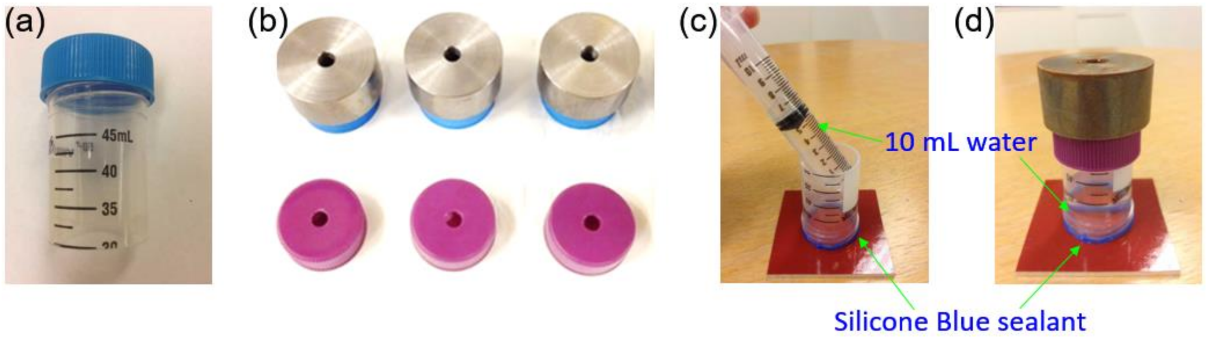

Figure S5. The ice mold components. (a) a cut 50 mL PP centrifuge tube; (b) metal cylinders and tube covers; (c) the injection of 10 mL water; (d) metal cylinder covered on the PP tube during ice formation.

Samples	Elastic modulus (GPa)	Hardness (GPa)
S1	4.312±0.175	1.572±0.134
S2	5.168±0.305	1.299±0.171
S3	3.841±0.423	1.066±0.152
S4	3.835±0.177	1.169±0.0542
S5	4.344±0.183	1.148±0.0785
S6	3.385±0.453	0.9389±0.132
S7	3.994±0.4089	1.220±0.162
S8	4.156±0.780	1.201±0.243
S9	3.747±0.0541	1.064±0.0302
S10	3.224±0.154	0.9750±0.0878
S11	4.156±0.0326	1.215±0.0362
S12	3.104±0.188	0.8860±0.106
S13	1.627±0.0243	0.3953±0.00930
S14	1.748±0.0709	0.3919±0.0312
S15	3.103±0.336	1.002±0.0408
S16	1.595±0.0359	0.3906±0.0402
S17	3.498±0.0507	0.7757±0.142
S18	6.067±0.375	0.3049±0.0178
S19	5.913±0.0885	0.1367±0.0214
S20	4.648±0.155	0.2477±0.00950
S21	5.823±0.861	0.411±0.116
S22	4.608±0.348	0.2778±0.0509
S23	73.66±2.63	3.819±0.124
S24	241.4±7.23	4.361±0.729

Table S2. The elastic modulus and hardness of S1-S24.

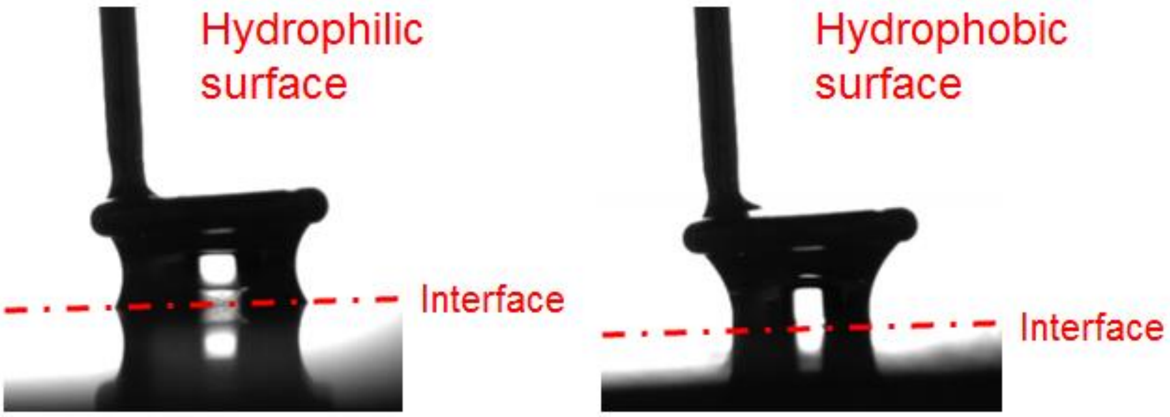

Figure S6. Captured photos during measurements of water adhesion forces.
